# Novel pyrazolo[3,4-*d*]pyrimidines: design, synthesis, anticancer activity, dual EGFR/ErbB2 receptor tyrosine kinases inhibitory activity, effects on cell cycle profile and caspase-3-mediated apoptosis

**DOI:** 10.1080/14756366.2018.1564046

**Published:** 2019-01-27

**Authors:** Mai Maher, Asmaa E. Kassab, Ashraf F. Zaher, Zeinab Mahmoud

**Affiliations:** Department of Pharmaceutical Organic Chemistry, Faculty of Pharmacy, Cairo University, Cairo, Egypt

**Keywords:** Pyrazolo[3,4-d]pyrimidines, design, synthesis, anticancer activity, EGFR, ErbB2, caspase-3, cell cycle arrest profile, apoptosis

## Abstract

A series of novel pyrazolo[3,4-*d*]pyrimidines was synthesised. Twelve synthesised compounds were evaluated for their anticancer activity against 60 human tumour cell lines by NCI (USA). Compound **7d** proved prominent anticancer activity. It showed 1.6-fold more potent anti-proliferative activity against OVCAR-4 cell line with IC_50_ = 1.74 μM. It also exhibited promising potent anticancer activity against ACHN cell line with IC_50_ value 5.53 μM, representing 2.2-fold more potency than Erlotinib. Regarding NCI-H460 cell line, compound **7d** (IC_50_ = 4.44 μM) was 1.9-fold more potent than Erlotinib. It inhibited EGFR and ErbB2 kinases at sub-micromolar level (IC_50_ = 0.18 and 0.25 µM, respectively). Dual inhibition of EGFR and ErbB2 caused induction of apoptosis which was confirmed by a significant increase in the level of active caspase-3 (11-fold). It showed accumulation of cells in pre-G1 phase and cell cycle arrest at G2/M phase.

## 1. Introduction

**Figure 1.**
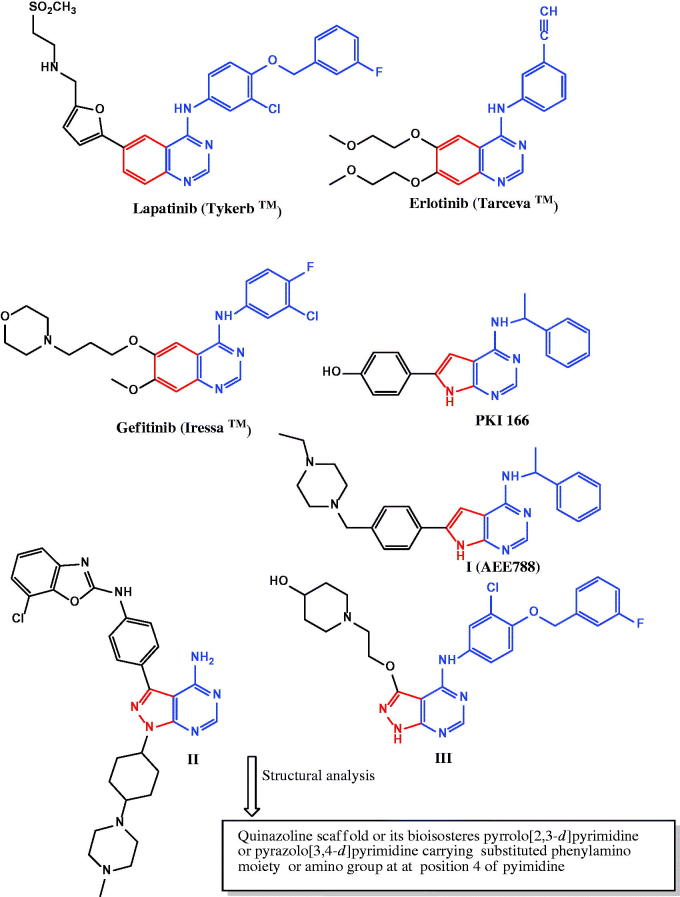
Examples of dual EGFR/ErbB2 inhibitors.

**Figure 2.**
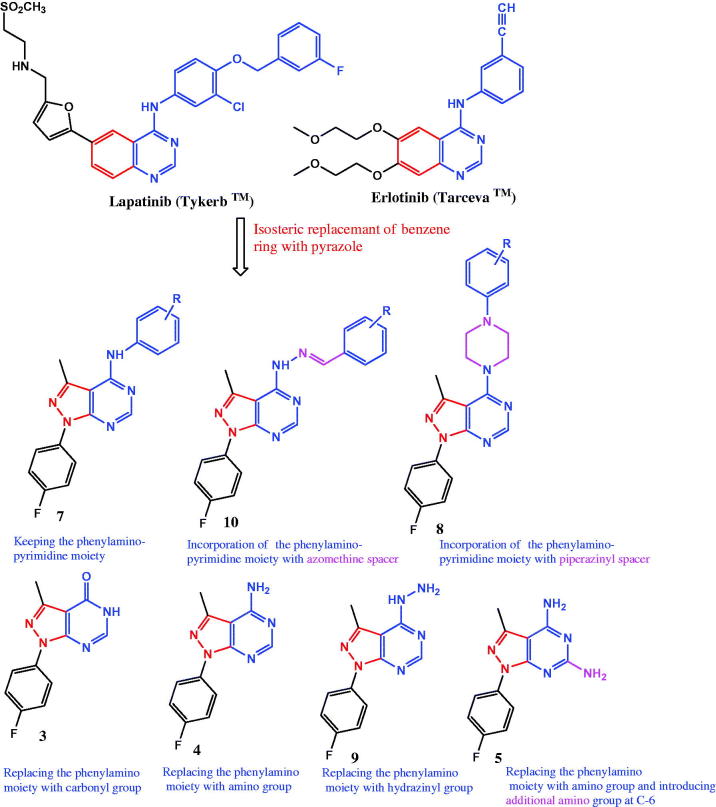
Design strategy for the target pyrazolo[3,4-*d*]pyrimidines.

The EGFR family comprises four distinct membrane tyrosine kinase receptors: (EGFR/ErbB1, HER2/ErbB2, HER3/ErbB3 and HER4/ErbB4) located in the plasma membrane and activated in response to ligand binding^1^^,^^2^. The interaction of EGFRs with the ligands causes receptor homo- and hetero-dimerisation, this interaction initiates a cascade of events that control diverse biological processes such as proliferation, differentiation, migration and apoptosis. Thus, deregulation of this transduction pathway has been implicated in many types of human cancers and is associated with poor clinical prognosis^2–5^. Moreover, members of EGFR family have been shown to be oncogenic. High expression levels of EGFR and ErbB2 has been implicated in the development of various types of cancers including breast, lung, colorectal, ovarian, prostate, head and neck^6–8^. EGFR and ErbB2 pathways are interconnected since both receptors have the highest homology among the EGFR family members in their kinase catalytic domains and share many similar biochemical and kinetic properties^9^. Additionally, the use of multiple ErbB receptors for cell proliferation and survival in tumour cells and the synergistic transforming effects of EGFR and ErbB2 lead to the hypothesis that targeting both the EGFR and ErbB2 catalytic domains simultaneously through dual EGFR/ErbB2 inhibition would have superior therapeutic effects relative to single-agent treatment for cancer^10^. Furthermore, a multi-targeted approach may improve the outcome of anti-EGFR therapies since the blockade of EGFR by EGFR tyrosine kinase inhibitors is insufficient to eradicate established tumors because of independently activated survival pathways^11^. Erlotinib (TarcevaTM)^12^, Gefitinib (IressaTM)^13^ and Lapatinib (Tykerb^TM^)^14^ (Figure 1) are low-molecular-weight dual inhibitors of EGFR/ErbB2 tyrosine kinases that compete with adenosine triphosphate (ATP) to block the catalytic domain of these receptors. They had been approved for the chemotherapeutic treatment of cancer patients. The main structural features of these drugs are quinazoline scaffold containing a substituted phenyl amino pyrimidine moiety. Purine is a heterocyclic nucleus which exists in the chemical architecture of various bioactive compounds. It is an important pharmacophore, which is widely used in the development of protein kinase inhibitors *via* introduction of substituents on the 2, 6 and 9 positions^15^. Pyrazolo[3,4-*d*]pyrimidine as a bioisostere of purine has drawn a considerable attention as a privileged scaffold for the design and discovery of novel anticancer agents^16^^,^^17^. In addition, pyrazolo[3,4-*d*]pyrimidine or its bioisostere pyrrolo[2,3-d]pyrimidine are common structural motifs of potent dual EGFR/ErbB2 inhibitors such as PKI166^18^^,^**I** (AEE788)^19^, **II**^20^ and **III**^21^ (Figure 1). Motivated by all these findings, we have designed and synthesised a series of pyrazolo[3,4-*d*]pyrimidines as novel small molecules targeting both EGFR and ErbB2 tyrosine kinases to be useful for treatment of cancer *via* inhibition of cell growth and induction of apoptosis. Our strategy was directed towards carrying out the chemical modifications on the general features of anilinoquinazoline scaffold to substantiate the effect of such modifications on the anticancer activity and to identify potent anticancer agents (Figure 2). Initially, we aimed to replace the benzene moiety in the quinazoline skeleton by an isostere (pyrazole one). This respected nucleus is always bearing a 4-fluorophenyl group at N1 since fluorinated compounds are one of the research hotspots in modern medicinal chemistry^22^. Moreover, incorporation of a fluorine atom provides compounds with enhanced both pharmacokinetic and physicochemical properties as compared to their non-fluorinated analogs^23^^,^^24^. The second modification focused on introducing various phenyl amino groups on the pyrimidine moiety. We have introduced unsubstituted phenyl amino group, phenyl amino group substituted with electron donating groups or phenyl amino group substituted with electron withdrawing groups. The third modification included incorporating the phenyl amino group to the pyrimidine nucleus through a spacer such as azomethine group or piperazinyl linker. In the fourth modification, we have focused on replacement of the phenyl amino group by small pharmacophoric moieties as carbonyl, amino, morpholine, 4-methylpiperazine or hydrazinyl groups. These groups at such position are well acknowledged for the anticancer activity of the fused pyrimidine rings^25^^,^^26^. Finally, additional amino group was introduced at C-6 position of pyrazolopyrimidine core. Twelve of the newly synthesised pyrazolopyrimidines were subjected to *in vitro* anticancer screening by the National Cancer Institute (USA) against 60 different human cell lines. The most potent compound was selected to be further studied through determination of its half maximal inhibitory concentration (IC_50_) values against ovarian cancer OVCAR-4, lung cancer NCI-H460, NCI-H226 and renal cancer ACHN cell lines. In order to explore the mechanistic pathways of the anticancer activity of **7d**, it was evaluated in EGFR, ErbB2 and active caspase-3 assays. Moreover, we also investigated its effect on the normal cell cycle profile and induction of apoptosis in the OVCAR-4 cell line.

## Experimental part

### General

All melting points were determined with Stuart SMP10 apparatus and the values given are uncorrected. IR spectra (KBr, cm^−1^) were determined on Shimadzu IR 8400 s spectrophotometer (Faculty of Pharmacy, Cairo University, Egypt).1H NMR and 13 C NMR spectra were recorded on Bruker 400-BB 400 MHz spectrometers (Microanalytical Unit, Faculty of Pharmacy, Cairo University, Egypt) using tetramethylsilane (TMS) as an internal standard. Chemical shift values are recorded in ppm on δ scale. Mass spectra were recorded on ISQLT single quadrapole mass spectrometer (at the Regional Center for Mycology and Biotechnology, Microanalytical Center, Al-Azhar University, Egypt). Elemental analyses were carried out at the Regional center for Mycology and Biotechnology, Faculty of Pharmacy, Al-Azhar University, Egypt. The reactions progression was monitored by TLC using aluminum sheets precoated with UV fluorescent silica gel (Merck 60 F 254) and visualised using UV lamp. Solvent system used was ethyl acetate, hexane with different ratios.

### 1–(4-Fluorophenyl)-3-methyl-1*H*-pyrazolo[3,4-*d*]pyrimidin-4(5*H*)-one (3)

A well-stirred solution of compound **2** (1.08 g, 0.005 mol) in formic acid (20 ml) was heated under reflux for 12 h. After cooling down, the formed precipitate was filtered, washed twice with water, dried and then recrystallised from absolute ethanol to afford **3**. Yield: 41%; mp: >300 °C; IR (KBr) cm^−1^: 3122 (NH str), 3055, 3010 (CH aromatic str), 2978, 2920, 2872 (CH aliphatic str), 1697 (C=O str), 1598, 1521 (C=N, C=C aromatic str); ^1^H NMR (DMSO-d_6_ ppm) δ: 2.52 (*s*, 3H, CH_3_), 7.36–7.40 (dd, 2H, *J* = 8.84 Hz), 8.03–8.05 (dd, 2H, *J* = 4.88 Hz, *J* = 9 Hz, ArH), 8.14 (*s*, 1H, C6-H), 12.36 (*s*, 1H, NH, D_2_O exchangeable); 13 C NMR (DMSO-d_6_ ppm) δ: 13.7 (CH_3_), 106.0, 116.27, 116.50 (d, *J* = 23 Hz), 123.81, 123.89 (d, *J* = 8 Hz) 124.0, 135.11, 135.13 (d, *J* = 2 Hz) 146.4, 149.5, (d, *J* = 308 Hz, C-F), 152.6, 158.4, 159.5 (Ar Cs), 161.9 (C=O); MS (m/z, %): 244 [M^.+^, 27.56], 95 [100.00](C_6_H_4_F^⌉+^). Anal. Calcd for C_12_H_9_FN_4_O (244.22): C, 59.01; H, 3.71; N, 22.94. Found: C, 59.17; H, 3.78; N, 23.12.

### 1–(4-Fluorophenyl)-3-methyl-1*H*-pyrazolo[3,4-*d*]pyrimidin-4-amine (4)

A solution of compound **2** (0.3 g, 0.001 mol) in formamide (10 ml) was stirred while heated under reflux for 4 h. The reaction mixture was cooled, the formed solid was filtered, washed with aqueous ethanol, dried and recrystallised from ethanol to give **4**. Yield: 68%; mp: >300 °C; IR (KBr) cm^−1^: 3498, 3394 (NH_2_ str), 3124, 3055 (CH aromatic str), 2924 (CH aliphatic str), 1597, 1450 (C=N, C=C aromatic str); ^1^H NMR (DMSO-d_6_ ppm) δ: 2.62 (*s*, 3H, CH_3_), 7.33–7.38 (dd, 2H, *J* = 8.80 Hz, *J* = 8.92 Hz, ArH), 7.47 (br *s*, 2H, NH_2_, D_2_O exchangeable), 8.16–8.21 (dd, 2H, *J* = 5 Hz, *J* = 9 Hz, ArH), 8.26 (*s*, 1H, C6-H); 13 C NMR (DMSO-d_6_ ppm) δ: 14.8 (CH_3_), 100.6, 116.11, 116.34 (d, *J* = 23 Hz), 122.55, 122.63 (d, *J* = 8 Hz), 135.79, 135.81 (d, *J* = 2 Hz), 143.3, 154.50, 157.06 (d, *J* = 265 Hz), 158.8, 159.0, 161.2 (Ar Cs); MS (m/z, %): 243 [M^.+^, 61.00], 95 [100.00] (C_6_H_4_F^⌉+^). Anal. Calcd for C_12_H_10_FN_5_ (243.24): C, 59.25; H, 4.14; N, 28.79. Found: C, 59.48; H, 4.16; N, 29.05.

### 1–(4-Fluorophenyl)-3-methyl-1*H*-pyrazolo[3,4-*d*]pyrimidine-4,6-diamine (5)

Guanidine HCl (2.1 g, 0.022 mol) was added to a solution of sodium (2.6 g, 0.113 mol) in methanol (95 ml), the precipitated sodium chloride was filtered off. Compound **2** (3.24 g, 0.015 mol) was added to the filtrate. The reaction mixture was heated under reflux for 8 h. The mixture was cooled to room temperature. The formed solid was filtered, washed with aqueous ethanol and recrystallised from ethanol to yield **5**.Yield: 83%; mp: >300 °C; IR (KBr) cm^−1^: 3392–3215 (2 NH_2_ str), 3001 (CH aromatic str), 2962, 2927, 2854 (CH aliphatic str), 1593, 1560, 1508 (C=N, C=C aromatic str); ^1^H NMR (DMSO-d_6_ ppm) δ: 2.37 (*s*, 3H, CH_3_), 7.29–7.38 (dd, 2H, *J* = 8.8 Hz, *J* = 8.9 Hz, ArH), 7.55 (*s*, 2H, NH_2_, D_2_O exchangeable), 8.05–8.08 (dd, 1H, *J* = 4.9 Hz, *J* = 8.9 Hz, ArH), 8.31–8.36 (dd, 1H, *J* = 4.9 Hz, *J* = 8.9 Hz, ArH), 8.34 (*s*, 2H, NH_2_, D_2_O exchangeable); 13 C NMR (DMSO-d_6_ ppm) δ: 13.8 (CH_3_), 104.0, 116.02, 116.25 (d, *J* = 23 Hz), 116.55, 116.77 (d, *J* = 22 Hz), 122.9, 127.01, 127.10 (d, *J* = 8 Hz), 135.8, 145.8, 159.2, 160.4, 167.4, 176.5 (Ar Cs); MS (*m*/*z*, %): 258 [M^.+^, 99.78], 43 [100.00] (CH_3_N^⌉+^). Anal. Calcd for C_12_H_11_FN_6_ (258.25): C, 55.81; H, 4.29; N, 32.54. Found: C, 56.04; H, 4.35; N, 32.79.

### 4-Chloro-1–(4-fluorophenyl)-3-methyl-1*H*-pyrazolo[3,4-*d*]pyrimidine (6)

Compound **3** (0.55 g, 0.0023 mol) was mixed with phosphorus oxychloride (80 ml). Ten drops of DMF was then added dropwise to this mixture. The resulting mixture was heated under reflux with stirring for 15 h. The reaction mixture was cooled, poured on ice-cold water (50 ml) with continuous stirring and sprinkling of sodium carbonate till neutralisation. The precipitated product was filtered, washed several times with aqueous ethanol, dried and recrystallised from absolute ethanol to afford **6**. Yield: 71%; mp: 178–180 °C; IR (KBr) cm^−1^: 3067 (CH aromatic str), 2950, 2916 (CH aliphatic str), 1577, 1550 (C=N, C=C aromatic str); ^1^H NMR (DMSO-d_6_ ppm) δ: 2.74 (s, 3H, CH_3_), 7.45 (d, 2H, ArH), 8.14 (d, 2H, ArH), 8.91 (*s*, 1H, C6-H); 13 C NMR (DMSO-d_6_ ppm) δ: 14.3 (CH_3_), 113.5, 116.57, 116.80 (d, *J* = 23 Hz), 123.7, 134.5, 140.3, 143.7, 153.60, 156.01 (d, *J* = 241 Hz, C-F), 156.4, 162.8 (Ar Cs). Anal. Calcd for C_12_H_8_ClFN_4_ (262.67): C, 54.87; H, 3.07; N, 21.33. Found: C, 55.12; H, 3.14; N, 21.59.

### General procedure for the preparation of compounds (7a–d)

A mixture of the chloro derivative **6** (0.3 g, 0.001 mol) and the selected aromatic amine (0.001 mol) was dispersed in isopropanol (2 ml). The reaction mixture was heated under reflux for 6 h. The reaction mixture was cooled. The obtained solid was filtered, dried and recrystallised from ethanol to afford **7a–d**.

### 1–(4-Fluorophenyl)-3-methyl-*N*-phenyl-1*H*-pyrazolo[3,4-*d*]pyrimidin-4-amine (7a)

Yield: 75%; mp: 164–166 °C; IR (KBr) cm^−1^: 3444 (NH str), 3082 (CH aromatic str), 2920, 2850 (CH aliphatic str), 1570, 1515, 1496 (C=N, C=C aromatic str); ^1^H NMR (DMSO-d_6_ ppm) δ: 2.78 (s, 3H, CH_3_), 7.16–7.20 (*t*, 1H, ArH), 7.37–7.43 (*m*, 4H, ArH), 7.70 (d, 2H, *J* = 8 Hz, ArH), 8.18–8.21 (dd, 2H, *J* = 4.9 Hz, *J* = 8 Hz, ArH), 8.43 (*s*, 1H, C6-H), 8.83 (*s*, 1H, NH, D_2_O exchangeable); 13 C NMR (DMSO-d_6_ ppm) δ: 15.1 (CH_3_), 101.8, 116.21, 116.43 (d, *J* = 22 Hz), 122.89, 122.97 (d, *J* = 8 Hz), 123.8, 124.7, 128.9, 135.5, 138.9, 142.9, 154.4, 156.0, 156.44, 159.07 (d, *J* = 263 Hz, C-F), 161.4 (Ar Cs); MS (*m*/*z*, %): 319 [M^.+^, 8.22], 71 [100.00]. Anal. Calcd for C_18_H_14_FN_5_ (319.34): C, 67.70; H, 4.42; N, 21.93. Found: C, 67.96; H, 4.39; N, 22.19.

### *N*,1-Bis(4-fluorophenyl)-3-methyl-1*H*-pyrazolo[3,4-*d*]-pyrimidin-4-amine (7b)

Yield: 75%; mp: 206–208 °C; IR (KBr) cm^−1^: 3444 (NH str), 3097, 3059 (CH aromatic str), 2916, 2850 (CH aliphatic str), 1593, 1566, 1512 (C=N, C=C aromatic str); ^1^H NMR (DMSO-d_6_ ppm) δ: 2.77 (*s*, 3H, CH_3_), 7.21–7.26 (dd, 2H, *J* = 8.8 Hz, *J* = 8.8 Hz, ArH), 7.35–7.39 (dd, 2H, *J* = 8.8 Hz, *J* = 8.9 Hz, ArH), 7.67–7.71 (dd, 2H, *J* = 5 Hz, *J* = 8.9 Hz, ArH), 8.16–8.20 (dd, 2H, *J* = 4.9 Hz, *J* = 7 Hz, ArH), 8.39 (*s*, 1H, C6-H), 8.85 (*s*, 1H, NH, D_2_O exchangeable); 13 C NMR (DMSO-d_6_ ppm) δ: 15.2 (CH_3_), 101.7, 115.38 115.60 (d, *J* = 22 Hz), 116.2, 116.46 (d, *J* = 22 Hz), 122.91, 122.99 (d, *J* = 8 Hz), 123.0, 126.09, 126.17, (d, *J* = 8 Hz) 135.2, 135.5, 143.0, 154.4, 156.1, 156.4, 158.20, 160.5 (d, *J* = 239 Hz, C-F), 159.08, 161.49 (d, *J* = 241 Hz, C-F) (Ar Cs); MS (m/z, %): 337 [M^.+^, 96.15], 336 [100.00] (M-H^⌉+^). Anal. Calcd for C_18_H_13_F_2_N_5_ (337.33): C, 64.09; H, 3.88; N, 20.76. Found: C, 64.18; H, 3.94; N, 20.98.

### 1–(4-Fluorophenyl)-3-methyl-*N*-(*p*-tolyl)-1*H*-pyrazolo[3,4-*d*]- pyrimidin-4-amine (7c)

Yield: 70%; mp: 203–205 °C; IR (KBr) cm^−1^: 3444 (NH str), 3012 (CH aromatic str), 2920, 2850 (CH aliphatic str), 1566, 1512, 1450 (C=N, C=C aromatic str); ^1^H NMR (DMSO-d_6_ ppm) δ: 2.32 (*s*, 3H, benzylic CH_3_), 2.76 (*s*, 3H, CH_3_), 7.22 (d, 2H, *J* = 8 Hz, ArH), 7.36–7.40 (dd, 2H, *J* = 8.8 Hz, *J* = 8.9 Hz, ArH), 7.56 (d, 2H, *J* = 8 Hz, ArH), 8.17 – 8.21 (dd, 2H, *J* = 4.9 Hz, *J* = 8.9 Hz, ArH), 8.39 (*s*, 1H, C6-H), 8.75 (*s*, 1H, NH, D_2_O exchangeable); 13 C NMR (DMSO-d_6_ ppm) δ: 15.2 (CH_3_), 21.0 (benzylic CH_3_), 101.6, 116.21, 116.43 (d, *J* = 22 Hz), 122.86, 122.94 (d, *J* = 8 Hz), 124.0, 129.3, 129.8, 133.9, 135.56, 135.59 (d, *J* = 3 Hz), 136.3, 142.9, 154.4, 156.1, 156.4, 159.05, 161.46 (d, *J* = 241 Hz, C-F) (Ar Cs); MS (*m*/*z*, %): 333 [M^.+^, 70.98], 71 [100.00]. Anal. Calcd for C_19_H_16_FN_5_ (333.36): C, 68.46; H, 4.84; N, 21.01. Found: C, 68.70; H, 4.91; N, 21.37.

### *N*-(3-Chloro-4-fluorophenyl)-1–(4-fluorophenyl)-3-methyl-1*H*-pyrazolo[3,4-*d*] pyrimidin-4-amine (7d)

Yield: 80%; mp: 234–236 °C; IR (KBr) cm^−1^: 3444 (NH str), 3100, 3016 (CH aromatic str), 2954, 2920, 2850 (CH aliphatic str), 1570, 1512, 1465 (C=N, C=C aromatic str); ^1^H NMR (DMSO-d_6_ ppm) δ: 2.78 (*s*, 3H, CH_3_), 7.36–7.40 (dd, 2H, *J* = 8.80 Hz, *J* = 8.84 Hz, ArH), 7.42–7.47 (dd, 1H, *J* = 9 Hz, *J* = 9.1 Hz, ArH), 7.70–7.74 (*m*, 1H, ArH), 7.99–8.01 (dd, 1H, *J* = 6.8 Hz, *J* = 2.4 Hz, ArH), 8.16–8.19 (dd, 2H, *J* = 4.9 Hz, *J* = 8.8 Hz, ArH), 8.46 (*s*, 1H, C6-H), 8.90 (*s*, 1H, NH, D_2_O exchangeable); 13 C NMR (DMSO-d_6_ ppm) δ: 15.2 (CH_3_), 101.8, 106.0, 116.26, 116.48 (d, *J* = 22 Hz), 116.82, 117.04 (d, *J* = 22 Hz), 119.13, 119.31 (d, *J* = 18 Hz), 122.97, 123.05 (d, *J* = 8 Hz), 123.82, 123.91 (d, *J* = 9 Hz), 124.19, 124.26 (d, *J* = 7 Hz), 125.3, 135.4, 136.2, 142.9, 153.07, 155.72 (d, *J* = 265 Hz, C-F), 154.37, 156.34 (d, *J* = 197 Hz) (Ar Cs); MS (*m/z*, %): 371 [M^.+^, 0.87], 258 [100.00]. Anal. Calcd for C_18_H_12_ClF_2_N_5_ (371.77): C, 58.15; H, 3.25; N, 18.84. Found: C, 58.43; H, 3.30; N, 19.12.

### General procedure for the preparation of compounds (8a–d)

Compound **6** (0.262 g, 0.001 mol) was added to the selected substituted amine (0.001 mol) and triethyl amine (0.1 g, 0.001 mol) in absolute ethanol (18 ml). The reaction mixture was heated under reflux for 15 h. After cooling down to room temperature, the precipitated product was filtered, dried and recrystallised from ethanol to obtain compounds **8a–d**.

### 1–(4-Fluorophenyl)-3-methyl-4-morpholino-1*H*-pyrazolo[3,4-*d*]pyrimidine (8a)

Yield: 75%; mp: 160–162 °C; IR (KBr) cm^−1^: 3041, 3014 (CH aromatic str), 2978, 2963, 2948, 2926 (CH aliphatic str), 1560, 1512, 1479 (C=N, C=C aromatic str); ^1^H NMR (DMSO-d_6_ ppm) δ: 2.62 (*s*, 3H, CH_3_), 3.74–3.78 (*m*, 8H, 4CH_2_ of morpholine), 7.35–7.40 (dd, 2H, *J* = 8.8 Hz, *J* = 8.9 Hz, ArH), 8.13–8.17 (dd, 2H, *J* = 4.9 Hz, *J* = 8.9 Hz, ArH), 8.40 (*s*, 1H, C6-H); 13 C NMR (DMSO-d_6_ ppm) δ: 17.4 (CH_3_), 48.7, 66.4 (4CH_2_ of morpholine), 102.4, 116.08, 116.31 (d, *J* = 23 Hz), 123.06, 123.14 (d, *J* = 8 Hz), 135.36, 135.39 (d, *J* = 3 Hz), 141.9, 155.0, 155.2, 159.09, 159.9, 161.51 (d, *J* = 242 Hz, C-F) (Ar Cs); MS (m/z, %): 313 [M^.+^, 7.01], 57 [100.00] (C_3_H_5_O^⌉+^). Anal. Calcd for C_16_H_16_FN_5_O (313.33): C, 61.33; H, 5.15; N, 22.35. Found: C, 61.71; H, 5.19; N, 22.52.

### 1–(4-Fluorophenyl)-3-methyl-4–(4-methylpiperazin-1-yl)-1*H*-pyrazolo[3,4-*d*]pyrimidine (8b)

Yield: 88%; mp: 253–255 °C; IR (KBr) cm^−1^: 3089, 3043 (CH aromatic str), 2951, 2920, 2850 (CH aliphatic str), 1558, 1516, 1458 (C=N, C=C aromatic str); ^1^H NMR (DMSO-d_6_ ppm) δ: 2.26 (*s*, 3H, N-CH_3_), 2.62 (*s*, 3H, CH_3_), 3.30–3.34 (*m*, 4H, 2CH_2_ of piperazine) 3.76–3.78 (*m*, 4H, 2CH_2_ of piperazine), 7.35–7.39 (dd, 2H, *J* = 8.8 Hz, *J* = 8.7 Hz, ArH), 8.13–8.16 (dd, 2H, *J* = 4.9 Hz, *J* = 8.7 Hz, ArH), 8.38 (*s*, 1H, C6-H); 13 C NMR (DMSO-d_6_ ppm) δ: 17.3 (CH_3_), 45.7 (N-CH_3_), 47.9 (2CH_2_ of piperazine), 54.5 (2CH_2_ of piperazine), 102.6, 116.10, 16.34 (d, *J* = 24 Hz)), 123.1, 135.3, 142.0, 149.7, 155.1, 155.2, 159.09, 159.9, 161.52 (d, *J* = 243 Hz, C-F) (Ar Cs); MS (m/z, %): 326 [M^.+^, 5.27], 256 [100.00] (M-70^⌉+^). Anal. Calcd for C_17_H_19_FN_6_ (326.37): C, 62.56; H, 5.87; N, 25.75. Found: C, 62.80; H, 5.94; N, 26.01.

### 4–(4-(4-Chlorophenyl)piperazin-1-yl)-1–(4-fluorophenyl)-3-methyl-1H-pyrazolo[3,4-*d*]pyrimidine (8c)

Yield: 80%; mp: 167–169 °C; IR (KBr) cm^−1^: 3043 (CH aromatic str), 2920, 2850 (CH aliphatic str), 1600, 1566, 1512, 1458 (C=N, C=C aromatic str); ^1^H NMR (DMSO-d_6_ ppm) δ: 2.67 (*s*, 3H, CH_3_), 3.20–3.35 (*m*, 8H, 4CH_2_ of piperazine), 6.99–7.03 (*m*, 2H, ArH), 7.07–7.11 (dd, 2H, *J* = 5 Hz, *J* = 9 Hz, ArH), 7.36–7.42 (*m*, 2H, ArH), 8.13–8.18 (dd, 2H, *J* = 5 Hz, *J* = 9 Hz, ArH), 8.42 (*s*, 1H, C6-H); 13 C NMR (DMSO-d_6_ ppm) δ: 17.5 (CH_3_), 45.6, 46.6, 48.1, 49.4 (4CH_2_ of piperazine), 102.6, 116.2 (d, C-F), 118.4, 123.3, 123.8, 135.1, 142.1, 143.2, 146.4, 148.0, 149.5, 152.6, 155.3, 156.6, 158.3, 159.9,164.5 (Ar Cs); MS (m/z, %): 422 [M^.+^, 0.95], 256 [100.00]. Anal. Calcd for C_22_H_20_ClFN_6_ (422.89): C, 62.48; H, 4.77; N, 19.87. Found: C, 62.75; H, 4.84; N, 19.65.

### 1–(4-Fluorophenyl)-4–(4-(4-methoxyphenyl)piperazin-1-yl)-3-methyl-1*H*-pyrazolo[3,4-*d*]pyrimidine (8d)

Yield: 96%; mp: 188–190 °C; IR (KBr) cm^−1^: 3082, 3059 (CH aromatic str), 2954, 2920, 2850 (CH aliphatic str), 1558, 1508, 1473 (C=N, C=C aromatic str); ^1^H NMR (DMSO-d_6_ ppm) δ: 2.68 (s, 3H, CH_3_), 3.20 (*t*, 4H, 2CH_2_ of piperazine), 3.70 (*s*, 3H, OCH_3_), 3.90 (*t*, 4H, 2CH_2_ of piperazine), 6.86 (d, 2H, *J* = 9 Hz, ArH), 6.97 (d, 2H, *J* = 9 Hz, ArH), 7.37 – 7.41 (*t*, 2H, *J* = 8.8 Hz, ArH), 8.15–8.18 (dd, 2H, *J* = 4.9 Hz, *J* = 9 Hz, ArH), 8.42 (*s*, 1H, C6-H); 13 C NMR (DMSO-d_6_ ppm) δ: 17.5 (CH_3_), 48.3 (OCH_3_), 50.2 (2CH_2_ of piperazine), 55.5 (2 CH_2_ of piperazine), 102.6, 108.0, 114.8, 116.18, 116.40 (d, *J* = 22 Hz), 118.3, 123.23, 123.31 (d, *J* = 8 Hz), 135.3, 138.2, 142.1, 145.5, 150.3, 153.75, 155.23 (d, *J* = 248 Hz, C-F), 155.3, 159.9 (Ar Cs); MS (*m*/*z*, %): 418 [M^.+^, 20.75], 256 [100.00]. Anal. Calcd for C_23_H_23_FN_6_O (418.47): C, 66.01; H, 5.54; N, 20.08. Found: C, 66.23; H, 5.60; N, 20.37.

### 1–(4-Fluorophenyl)-4-hydrazinyl-3-methyl-1*H*-pyrazolo[3,4-*d*]- pyrimidine (9)

A mixture of compound **6** (0.289 g, 0.0011 mol) and hydrazine hydrate (99%, 0.6 g, 0.012 mol) in absolute ethanol (20 ml) was heated under reflux with stirring for 8 h. The obtained solution was left to cool down. The produced precipitate was filtered, dried and recrystallised from 96% ethanol to yield **9**. Yield: 69%; mp: 208–210 °C; IR (KBr) cm^−1^: 3309–3120 (NH, NH_2_ str), 3047, 3008 (CH aromatic str), 2962, 2850 (CH aliphatic str), 1593, 1512 (C=N, C=C aromatic str); ^1^H NMR (DMSO-d_6_ ppm) δ: 2.62 (*s*, 3H, CH_3_), 4.80 (*s*, 2H, NH_2_, D_2_O exchangeable), 7.32–7.38 (dd, 2H, *J* = 7 Hz, *J* = 9 Hz, ArH), 8.14–8.19 (dd, 2H, *J* = 5 Hz, *J* = 9 Hz, ArH), 8.35 (*s*, 1H, C6-H), 8.90 (*s*, 1H, NH, D_2_O exchangeable); 13 C NMR (DMSO-d_6_ ppm) δ: 15.4 (CH_3_), 99.7, 116.11, 116.33 (d, *J* = 22 Hz), 122.76, 122.84 (d, *J* = 8 Hz), 135.70, 135.72 (d, *J* = 2 Hz), 142.8, 153.8, 156.49, 158.2, 158.94 (d, *J* = 245 Hz, C-F), 161.3 (Ar Cs); MS (*m*/*z*, %): 258 [M^.+^, 100.00]. Anal. Calcd for C_12_H_11_FN_6_ (258.25): C, 55.81; H, 4.29; N, 32.54. Found: C, 55.98; H, 4.37; N, 32.82.

### General procedure for the preparation of compounds (10a–d)

Five drops of glacial acetic acid were added to a solution of compound **9** (0.774 g, 0.003 mol) and the selected aromatic aldehyde (0.003 mol) in absolute ethanol (20 ml). The reaction mixture was heated under reflux for 6 h. After cooling the formed precipitate was filtered, dried and recrystallised from ethanol to give **10a–d**.

### 1–(4-Fluorophenyl)-4–(2-(methoxybenzylidene)hydrazinyl)-3-methyl-1*H*-pyrazolo[3,4-*d*]pyrimidine (10a)

Yield: 43%; mp: 266–268 °C; IR (KBr) cm^−1^: 3394 (NH str), 3062, 3001 (CH aromatic str), 2916, 2846 (CH aliphatic str), 1508, 1462 (C=N, C=C aromatic str); ^1^H NMR (DMSO-d_6_ ppm) δ: 2.67 (*s*, 3H, CH_3_), 3.86 (*s*, 3H, OCH_3_), 7.05 (d, 2H, *J* = 8 Hz, ArH), 7.38–7.42 (*t*, 2H, *J* = 8.8 Hz, ArH), 7.87–7.90 (*m*, 2H, ArH), 8.02–8.07 (*m*, 2H, ArH), 8.19 (*s*, 1H, N=CH), 8.21 (*s*, 1H, NH, D_2_O exchangeable), 8.47 (*s*, 1H, C6-H); 13 C NMR (DMSO-d_6_ ppm) δ: 14.8 (CH_3_), 55.8 (OCH_3_), 106.1, 111.6, 114.6, 116.23, 116.46 (d, *J* = 23 Hz), 121.3, 123.73, 123.80 (d, *J* = 7 Hz), 127.6, 128.0, 129.9, 132.1, 135.1, 138.9, 145.0, 149.5, 155.3, 159.40, 161.42 (d, *J* = 202 Hz, C-F) (Ar Cs and N=CH); MS (*m*/*z*, %): 376 [M^.+^, 64.38], 243 [100.00] (M-133^⌉+^). Anal. Calcd for C_20_H_17_FN_6_O (376.39): C, 63.82; H, 4.55; N, 22.33. Found: C, 64.07; H, 4.59; N, 22.17.

### 1–(4-Fluorophenyl)-4–(2-(4-hydroxy-3-methoxybenzylidene)- hydrazinyl)-3-methyl-1*H*-pyrazolo[3,4-*d*]pyrimidine (10b)

Yield: 75%; mp: 221–223 °C; IR (KBr) cm^−1^: 3510 (OH str), 3444 (NH str), 3055, 3005 (CH aromatic str), 2920, 2850 (CH aliphatic str), 1600, 1573, 1516 (C=N, C=C aromatic str); ^1^H NMR (DMSO-d_6_ ppm) δ: 2.86 (*s*, 3H, CH_3_), 3.83 (*s*, 3H, OCH_3_), 6.84–6.89 (dd, 2H, *J* = 4.7 Hz, *J* = 8 Hz, ArH), 7.29–7.34 (dd, 2H, *J* = 4.7 Hz, *J* = 8 Hz, ArH), 7.42 (*s*, 1H, ArH), 7.92–7.95 (*m*, 2H, ArH), 8.25 (*s*, 1H, N=CH), 8.52 (*s*, 1H, C6-H), 9.55 (br s, 1H, NH, D_2_O exchangeable), 11.84 (*s,* 1H, OH, D_2_O exchangeable); 13 C NMR (DMSO-d_6_ ppm) δ: 14.5 (CH_3_), 55.9 (OCH_3_), 110.5, 111.3, 115.79, 115.97 (d, *J* = 18 Hz), 116.2, 123.1, 123.80, 123.92 (d, *J* = 12 Hz), 124.5, 125.9, 127.1, 135.2, 138.8, 145.3, 148.44, 149.4, 150.44 (d, *J* = 200 Hz, C-F), 161.0 (Ar Cs and N=CH); MS (*m*/*z*, %): 392 [M^.+^, 22.72], 300 [100.00]. Anal. Calcd for C_20_H_17_FN_6_O_2_ (392.39): C, 61.22; H, 4.37; N, 21.42. Found: C, 61.43; H, 4.45; N, 21.80.

### 1–(4-Fluorophenyl)-3-methyl-4–(2-(3,4,5-trimethoxybenzylidene)- hydrazinyl)-1*H*-pyrazolo[3,4-*d*]pyrimidine (10c)

Yield: 69%; mp: 222–224 °C; IR (KBr) cm^−1^: 3421 (NH str), 3062 (CH aromatic str), 2920, 2850 (CH aliphatic str), 1581, 1504, 1454 (C=N, C=C aromatic str); ^1^H NMR (DMSO-d_6_ ppm) δ: 2.67 (*s*, 3H, CH_3_), 3.85 (*s*, 6H, 2 OCH_3_), 3.86 (*s*, 3H, CH_3_O), 7.22 (*s*, 2H, ArH), 7.36–7.41 (dd, 2H, *J* = 5.2 Hz, *J* = 8.9 Hz, ArH), 8.08–8.10 (*m*, 3H, ArH + NH), 8.31 (*s*, 1H, N=CH), 8.66 (*s*, 1H, C6-H); 13 C NMR (DMSO-d_6_ ppm) δ: 14.7 (CH_3_), 56.4 (2 OCH_3_), 60.63 (OCH_3_), 106.0, 110.9, 116.18, 116.40 (d, *J* = 22 Hz), 121.20, 123.63 (d, *J* = 243 Hz, C-F), 124.0, 128.3, 129.7, 132.3, 133.4, 135.2, 136.4, 138.3, 140.6, 141.6, 150.1, 153.6, 161.6 (Ar Cs and N=CH); MS (*m*/*z*, %): 436 [M^.+^, 3.40], 269 [100.00] (M-167^⌉+^). Anal. Calcd for C_22_H_21_FN_6_O_3_ (436.44): C, 60.54; H, 4.85; N, 19.26. Found: C, 60.82; H, 4.91; N, 19.38.

### 1–(4-Fluorophenyl)-3-methyl-4–(2-(pyridin-4-yl-methylidene) hydrazinyl)-1*H*-pyrazolo[3,4-*d*]pyrimidine (10d)

Yield: 51%; mp: 262–264 °C; IR (KBr) cm^−1^: 3433 (NH str), 3070, 3039 (CH aromatic str), 2920, 2850 (CH aliphatic str), 1562, 1516, 1473 (C=N, C=C aromatic str); ^1^H NMR (DMSO-d_6_ ppm) δ: 2.58 (*s*, 3H, CH_3_), 7.36–7.42 (dd, 2H, *J* = 4.9 Hz, *J* = 9.2 Hz, ArH), 7.92 (d, 2H, *J* = 5.8 Hz, pyridyl H), 8.01–8.05 (dd, 2H, *J* = 4.9 Hz, *J* = 9.2 Hz, ArH), 8.19 (*s*. 1H, N=CH), 8.44 (*s*, 1H, C6-H), 8.65 (d, 2H, *J* = 5.8 Hz, pyridyl H), 12.15 (*s*, 1H, NH, D_2_O exchangeable); 13 C NMR (DMSO-d_6_ ppm) δ: 14.5 (CH_3_), 102.5, 116.23, 116.46 (d, *J* = 23 Hz), 121.4, 122.0, 123.79, 123.88 (d, *J* = 9 Hz), 135.1, 142.7, 145.68, 148.41 (d, *J* = 273 Hz, C-F), 149.8, 150.1, 150.4, 150.8, 151.3, 155.5, 159.5 (Ar Cs and N=CH); MS (m/z, %): 347 [M^.+^, 100.00]. Anal. Calcd for C_18_H_14_FN_7_ (347.35): C, 62.24; H, 4.06; N, 28.23. Found: C, 62.52; H, 4.17; N, 28.61.

## Anticancer activity

### Measurement of anticancer activity against a panel of 60 cell lines

Anticancer activity screening of the newly synthesised compounds was measured *in vitro* utilising 60 different human tumour cell lines provided by US National Cancer Institute according to previously reported standard procedure^27–29^ as follows: Cells are inoculated into 96-well microtitre plates in 100 ml. After cell inoculation, the microtitre plates are incubated at 37 °C, 5% CO_2_, 95% air and 100% relative humidity for 24 h prior to addition of experimental compounds. After 24 h, two plates of each cell line are fixed *in situ* with TCA, to represent a measurement of the cell population for each cell line at the time of drug addition (Tz). Experimental compounds are solubilised in dimethyl sulphoxide at 400-fold the desired final maximum test concentration and stored frozen prior to use. At the time of compound addition, an aliquot of frozen concentrate is thawed and diluted to twice the desired final maximum test concentration with complete medium containing 50 mg/mL gentamicin. Aliquots of 100 ml of the compounds dilutions are added to the appropriate microtitre wells already containing 100 ml of medium, resulting in the required final compound concentration. Following compound addition, the plates are incubated for an additional 48 h at 37 °C, 5% CO_2_, 95% air, and 100% relative humidity. For adherent cells, the assay is terminated by the addition of cold trichloroacetic acid (TCA). Cells are fixed *in situ* by the gentle addition of 50 ml of cold 50% (w/v) TCA (final concentration, 10% TCA) and incubated for 60 min at 4 °C. The supernatant is discarded, and the plates are washed five times with tap water and air-dried. Sulphorhodamine B (SRB) solution (100 ml) at 0.4% (w/v) in 1% acetic acid is added to each well, and plates are incubated for 10 min at room temperature. After staining, unbound dye is removed by washing five times with 1% acetic acid and the plates are air-dried. Bound stain is subsequently solubilised with 10 mM trizma base, and the absorbance is read on an automated plate reader at a wavelength of 515 nm. For suspension cells, the methodology is the same except that the assay is terminated by fixing settled cells at the bottom of the wells by gently adding 50 ml of 80% TCA (final concentration, 16% TCA). Using the absorbance measurements [time zero, (Tz), control growth, (C), and test growth in the presence of compound (Ti)], the percentage growth is calculated for each compound. Percentage growth inhibition is calculated as:

[(Ti – Tz)/(C – Tz)] x 100 for concentrations for which Ti >/=Tz.

[(Ti – Tz)/Tz] x 100 for concentrations for which Ti < Tz.

## Measurement of IC_50_ against lung cancer NCI-H460, NCI-H226, ovarian cancer OVCAR-4 and renal cancer ACHN cell lines

### Cell culture

Cell Line cells were obtained from American Type Culture Collection, cells were cultured using Dulbecco's Modified Eagle's medium (DMEM) (Invitrogen/Life Technologies) supplemented with 10% foetal bovine serum (FBS) (Hyclone), 10 *μ*g/mL of insulin (Sigma), and 1% penicillin-streptomycin. All of the other chemicals and reagents were from Sigma, or Invitrogen. Plate cells (cells density 1.2 – 1.8 × 10,000 cells/well) in a volume of 100 *µ*L complete growth medium and 100 *μ*L of the tested compound per well in a 96-well plate for 24 h before the MTT assay.

### Cell culture protocol

Remove culture medium to a centrifuge tube. Briefly rinse the cell layer with 0.25% (w/v) Trypsin 0.53 *μ*M ethylenediaminetetraacetic acid (EDTA) solution to remove all traces of serum which contains Trypsin inhibitor. Add 2.0 to 3.0 ml of Trypsin EDTA solution to flask and observe cells under an inverted microscope until cell layer is dispersed. Add 6 to 8 ml of complete growth medium and aspirate cells by gently pipetting. Transfer the cell suspension to the centrifuge tube with the medium and centrifuge for 5 to 10 min. Discard the supernatant. Resuspend the cell pellet in fresh growth medium. Add appropriate aliquots of the cell suspension to new culture vessels. Incubate cultures at 37 °C for 24 h. After treatment of cells with the serial concentrations of the compound to be tested incubation is carried out for 48 h at 37 °C, then the plates are to be examined under the inverted microscope and proceed for the MTT assay.

### MTT assay protocol

The 3–(4, 5-dimethylthiazol-2-yl)-2,5-diphenyltetrazolium bromide (MTT) method^30^ of monitoring *in vitro* cytotoxicity is well suited for use with multiwell plates. The assessment of cell population growth is based on the capability of living cells to reduce the yellow product MTT to a blue product, formazan, by a reduction reaction occurring in the mitochondria. The five cell lines were incubated for 24 h in 96-microwell plates. The number of living cells in the presence or absence (control) of the various test compounds is directly proportional to the intensity of the blue colour, measured by spectrophotometry using (ROBONIK P2000 Spectrophotometer) at a wavelength of 570 nm. Measure the background absorbance of multiwell plates at 690 nm and subtract from the 570 nm measurement. Five concentrations ranging from 0.01 *μ*M to 100 *μ*M (with semi-log decrease in concentration) were tested for each of the compounds under study. Each experiment was carried out in triplicate. The IC_50_ values [the concentration required for 50% inhibition of cell viability] were calculated using sigmoidal dose response curve-fitting models.

### Measurement of inhibitory activity against EGFR

Compound **7d** was selected to be evaluated against EGFR enzyme using EnzyChrom^TM^ Kinase Assay Kit (EKIN-400) according to manufacturer’s instructions. In brief, set up 20 μL reaction mixture containing the EGFR kinase, ATP and substrate in the provided assay buffer. Set up a blank control that contains ATP and substrate but no enzyme. Incubate at desired temperature for 30 min. Prepare 900 μL 10 μM (adenosine diphosphate) ADP premix by mixing 3 μL 3 mM standard and 897 μL distilled water. Transfer 20 μL standards into separate wells of the plate. Prepare enough working reagent for each well. Add 40 μL working reagent to each assay well. Tap plate to mix. Incubate at room temperature for 10 min. Read fluorescence intensity at *λ*_exc_ = 530 nm and *λ*_em_ = 590 nm.Calculate kinase activity.

### Measurement of inhibitory activity against ErbB2

Compound **7d** was evaluated against ErbB2 enzyme using HTScan® HER2/ErbB2 Kinase Assay Kit according to manufacturer’s instructions. In brief, add 10 μL of 10 mM ATP to 1.25 ml of 6 μM substrate peptide. Dilute the mixture with dH_2_0 to 2.5 ml. Transfer enzyme from −80 °C to ice. Allow enzyme to thaw on ice. Micro-centrifuge briefly at 4^0^ C. Return immediately to ice. Add 10 μL of dithiothreitol (DTT) (1.25 M) to 2.5 ml of HTScan^®^ tyrosine kinase buffer. Transfer 1.2 ml of DTT/Kinase buffer to each enzyme. Incubate 12.5 μL of the reaction cocktail with 12.5 μL/well of pre-diluted compound of interest (10 μM) for 5 min at room temperature. Add 25 μL of ATP/substrate cocktail to 25 μL/well pre-incubated reaction cocktail/compound. Incubate reaction plate at room temperature for 30 min. Add 50 μL/well stop buffer to stop the reaction. Transfer 25 μL of each reaction and 75 μL dH_2_O/well to a 96-well streptavidin coated, plate and incubate at room temperature for 60 min. Wash three times with 200 μL/well phosphate-buffered saline (PBS). Dilute primary antibody, phospho-tyrosine mAb (P-Tyr-100), 1:1000 in PBS with 1% bovine serum albumin (BSA). Add 100 μL/well primary antibody. Incubate at room temperature for 60 min and wash three times. Prepare appropriate dilution of horseradish peroxidase (HRP) labelled secondary antibody in PBS with 1% BSA. Add 100 μL/well secondary antibody solution. Incubate at room temperature for 30 min and wash five times. Add 100 μL/well 3,3′,5,5′-tetramethylbenzidine (TMB) substrate. Incubate at room temperature for 15 min. Add 100 μL/well of stop solution. Mix well and read the absorbance at 450 nm with a microtitre plate reader.

### Measurement of the effect of compound 7d on the level of caspase-3 protein (Marker of apoptosis):

The level of human active caspase-3 protein was evaluated using Invitrogen (Catalog KHO1091) ELISA kit. The manufacturer’s instructions were followed in the following procedures. Add 100 μL of the standard diluent buffer to the zero standard wells. Add 100 μL of standards and controls or diluted samples to the appropriate microtitre wells. Cover wells with and incubate for 2 h at room temperature. Thoroughly aspirate or decant solution from wells and discard the liquid. Pipette 100 μL of caspase-3 (active) detection antibody solution into each well. Cover plate and incubate for 1 h at room temperature. Add 100 μL Anti-Rabbit IgG HRP working solution to each well. Cover wells with the plate cover and incubate for 30 min at room temperature. Add 100 μL of stabilised chromogen to each well. The liquid in the wells will begin to turn blue. Incubate for 30 min at room temperature. Stop solution has been added to each well. The solution in the wells should change from blue to yellow. Read the plate within 2 h after adding the stop solution. Use a curve fitting software to generate the standard curve.

### Cell cycle analysis of compound 7d

The OVCAR-4 cells were treated with compound **7d** at its IC_50_ concentration for 24 h. After treatment, the cells were washed twice with ice-cold PBS, collected by centrifugation, and fixed in ice-cold 70% (v/v) ethanol, washed with PBS, re-suspended with 0.1 mg/mL RNase, stained with 40 mg/mL propidium iodide (PI), and analysed by flow cytometry using FACS Calibur (Becton Dickinson)^31^. The cell cycle distributions were calculated using Cell-Quest software (Becton Dickinson). Exposure of OVCAR-4 cells to compound **7d** resulted in an interference with the normal cell cycle distribution as indicated.

### Annexin V-FITC assay

Annexin V fluorescein isothiocyanate (FITC)*/*PI apoptosis detection kit (BD Biosciences) was used according to the manufacturer’s instructions to measure the apoptotic activity of compound **7d**. OVCAR-4 cells were incubated with Annexin V-FITC. Apoptosis was induced by addition of compound **7d** at its IC_50_ to 4 × 10^6^ cell/T 75 flask and incubation for 24 h. The cells then were collected by trypsinisation and 0.5 × 10^6^ cells were washed twice with PBS and stained with 5 μL Annexin V-FITC and 5 μL PI in 1 × binding buffer for 15 min at room temperature in the dark. Analyses were performed using FACS Calibur flow cytometer (BD Biosciences, San Jose, CA).

## Results and discussion

### Chemistry

**Scheme 1.**
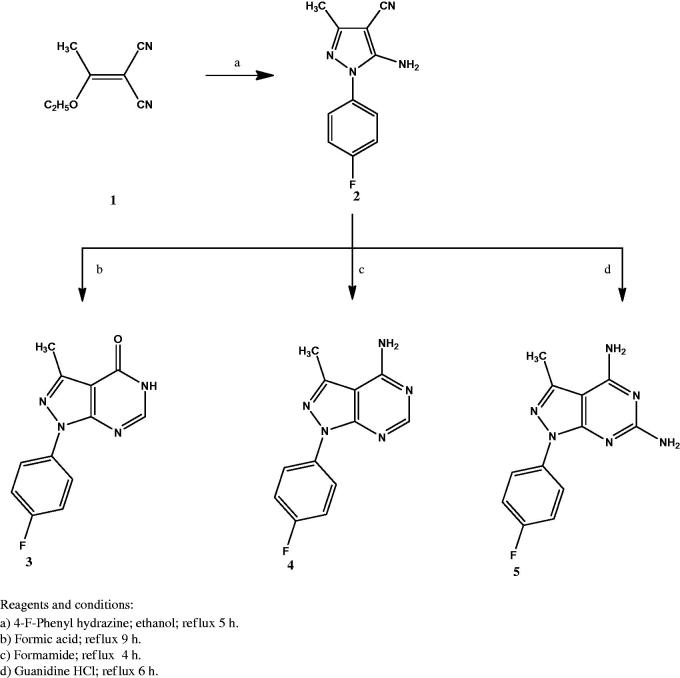
The synthetic path and reagents for the preparation of compounds **1–5**.

**Scheme 2.**
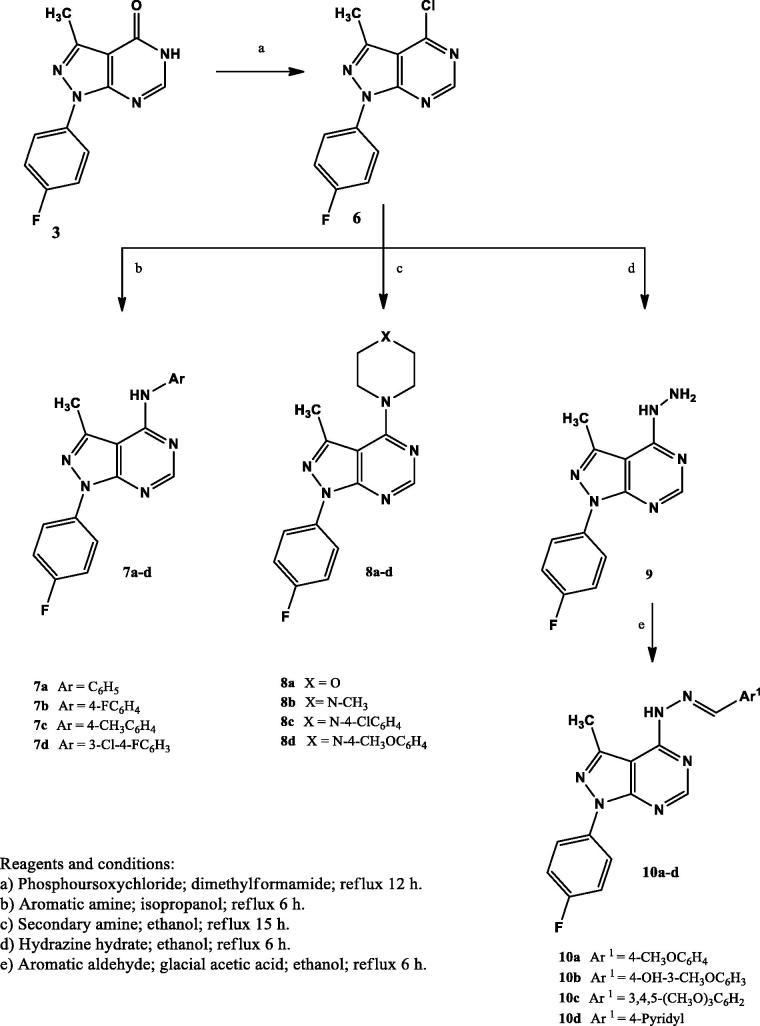
The synthetic path and reagents for the preparation of compounds **6–10**.

The synthesis of the new compounds is illustrated in Schemes 1 and 2. The preparation of the starting material **1** was achieved *via* heating triethyl orthoacetate and malononitrile in acetic anhydride as previously reported^32^. Compound **2**^33^ was prepared by refluxing 2–(1-ethoxyethylidene) malononitrile (**1**) with 4-fluorophenylhydrazine hydrochloride in absolute ethanol in the presence of triethylamine. Heating under reflux the amino cyano derivative **2** in formic acid afforded compound **3**. The IR spectrum of compound **3** was useful in tracing the disappearance of C≡N and NH_2_ absorption bands, while it showed the absorption band at 1697 cm^−1^ due to C=O group. The ^1^H NMR spectrum displayed the presence of a singlet signal at δ 8.14 ppm due to C6-H. In addition to the appearance of an exchangeable singlet signal at δ 12.36 ppm corresponding to NH proton. The 13C NMR spectrum pointed the formation of compound **3** by the appearance of C=O carbon at δ 161.9 ppm. Compound **4** was prepared in a good yield through the condensation of the amino cyano derivative **2** with formamide. The IR spectrum confirmed the disappearance of the C≡N group of the respective compound **2**. Furthermore, the ^1^H NMR spectrum showed an exchangeable singlet signal at δ 7.47 ppm corresponding to the NH_2_ protons, in addition to a singlet signal at δ 8.26 ppm indicating the C6-H proton of the pyrimidine ring. Reacting 5-amino-1–(4-fluorophenyl)-3-methyl-1*H*-pyrazole-4-carbonitrile (**2**) with guanidine in methanol gave the target compound **5**. The IR spectrum showed the appearance of bands at 3392 – 3215 cm^−1^ attributed to two NH_2_ groups. In accordance, the ^1^H NMR spectrum of compound **5** displayed two D_2_O exchangeable signals at δ 7.55 and 8.34 ppm attributed to 2-amino and 4-amino protons with the appropriate integration. The chloro derivative **6** was prepared by the reaction of the pyrazolopyrimidone **3** with phosphorus oxychloride. The IR spectrum lacked the absorption bands corresponding to NH and C=O groups at 3122 cm^−1^ and 1697 cm^−1^, respectively. Further structural evidence stemmed from the ^1^H NMR spectrum was the disappearance of the signal of NH proton. The reaction of the 4-chloropyrazolo[3,4-*d*]pyrimidine **6** with the appropriate aromatic amine in isopropyl alcohol afforded compounds **7a–d**. The IR spectra of **7a–d** showed the presence of an absorption band at 3444 cm^−1^ corresponding to NH group. The ^1^H NMR spectra supported the formation of **7a–d** through the appearance of the D_2_O exchangeable singlet signal at δ 8.75 – 8.90 ppm. The appearance of extra aromatic protons corresponding to substitution on the 4-amino moiety was another significant proof of the success of the reaction. The synthesis of the target compounds **8a–d** was accomplished through the reaction of the 4-chloropyrazolo[3,4-*d*]pyrimidine derivative **6** with the selected secondary amine in ethanol in the presence of a catalytic amount of triethylamine. The ^1^H NMR spectra of these compounds showed the presence of the 4 CH_2_ protons of the morpholinyl or piperazinyl moiety at δ 3.20 – 3.78 ppm, while the 13C NMR spectra indicated the presence of 4 CH_2_ carbons at δ 45.6 – 66.4 ppm. The hydrazinyl derivative **9** was obtained in a good yield *via* reacting the 4-chloropyrazolo[3,4-*d*]pyrimidine **6** with hydrazine hydrate in ethanol. The IR spectrum of compound **9** showed two absorption bands at the range 3309 – 3120 cm^−1^ indicating the presence of both NH and NH_2_ groups. The ^1^H NMR spectrum revealed the presence of two D_2_O exchangeable singlet signals at δ 4.80 and 8.90 ppm with proper integration, corresponding to NH_2_ and NH protons, sequentially. Condensation of the 4-hydrazinyl derivative **9** with the selected aromatic aldehyde in ethanol in the presence of glacial acetic acid yielded compounds **10a–d** in good yields. The ^1^H NMR of the target compounds revealed the appearance of a singlet signal at the range of δ 8.19 – 8.31 ppm corresponding to N=CH proton, in addition to additional characteristic aromatic protons denoting the success of the condensation reaction.

### Growth inhibition against a panel of 60 human tumor cell lines

In this study, 12 of the newly synthesised pyrazolopyrimidines were subjected to *in vitro* anticancer screening by the National Cancer Institute (USA) against 60 different human cell lines including (leukemia, non-small cell lung cancer, colon cancer, CNS cancer, melanoma, ovarian cancer, renal cancer, prostate cancer and breast cancer). The selected compounds were evaluated at single dose (1 0 ^−5 ^M). The growth inhibition percentages obtained from the single dose test for the selected compounds are shown in Tables 1 and 2. The analysis of the obtained *in vitro* data revealed that two lung cell lines NCI-H226 and NCI-H460 were sensitive to compound **7d** with growth inhibition percentages 62.91 and 58.46, sequentially. Compound **7d** also displayed potent cytotoxic activity against renal cell line ACHN with growth inhibition percentage 60.41 and against ovarian cancer cell line OVCAR-4 with growth inhibition percentage 53.12. Compound **6** showed promising selectivity against lung cancer cell line NCI-H522 with growth inhibition percentage 58.95. Other test compounds exhibited no activity against most investigated cell lines.

**Table 1.** Growth inhibition percentages obtained from the single dose (10^−5 ^M) test for compounds **3–6, 7c, d**.

Panel/cell line	Compound					
	**3**	**4**	**5**	**6**	**7c**	**7d**
*Leukemia*						
CCRF-CEM	−1.13	−12.67	−16.80	1.47	−8.18	0.70
HL-60(TB)	6.74	7.87	8.85	13.97	16.02	14.97
K-562	1.69	1.98	−11.90	15.44	0.26	1.78
MOLT-4	3.30	0.58	−0.48	13.75	1.83	4.27
RPMI-8226	−10.17	−17.49	−3.69	2.21	−4.47	9.77
SR	−1.58−	3.35	−1.68	14.42	6.02	25.37
*Non-Small Cell Lung Cancer*						
A549/**ATCC**	−2.93	−4.88	−1.96	11.58	3.08	40.51
EKVX	36.18	19.10	6.25	9.08	3.39	14.46
HOP-62	−6.53	−8.24	−8.35	15.44	−14.49	38.70
HOP-92	10.32	−1.54	1.64	7.91	3.79	22.78
NCI-H226	−9.27	−7.40	−8.08	15.05	−10.21	**62.91**
NCI-H23	4.09	4.73	11.79	7.86	4.63	7.53
NCI-H322M	5.19	7.26	8.14	5.32	10.04	12.02
NCI-H460	−9.62	−9.30	−18.78	−0.07	−9.33	**58.46**
NCI-H522	4.92	6.49	2.08	**58.95**	10.98	10.44
*Colon Cancer*						
COLO 205	−14.86	−18.39	−13.53	−14.88	−13.30	−3.96
HCC-2998	−2.16	−2.52	3.79	−5.66	1.34	1.79
HCT-116	−1.64	−8.66	−2.75	7.98	3.20	19.23
HCT-15	−6.88	−4.62	−3.69	1.32	−0.49	2.75
HT29	3.83	3.34	−6.50	9.55	11.70	14.38
KM12	1.48	3.50	1.24	0.99	−1.11	1.96
SW-620	−12.29	−18.66	−16.79	−2.88	−8.11	11.14
*CNS Cancer*						
SF-268	−1.71	3.71	−2.30	−1.16	1.06	3.94
SF-295	−4.14	−3.53	−1.89	−4.11	−3.83	39.96
SF-593	−0.99	−1.06	2.81	−7.81	−1.94	22.58
SNB-19	−3.69	−9.19	−7.94	7.79	7.21	20.45
SNB-75	0.29	8.18	5.33	6.36	−5.20	25.04
U251	−1.70	−7.56	−6.63	5.15	2.65	24.02
*Melanoma*						
LOX IMVI	2.19	1.75	2.19	6.64	−0.99	8.68
MALME-3M	−8.22	5.86	−7.80	−4.30	1.70	1.56
M14	−7.65	−3.62	−2.93	5.89	0.75	1.01
MDA-MB-435	−7.05	−5.15	−7.73	−8.83	−6.94	0.10
SK-MEL-2	−5.10	1.74	3.86	10.97	10.95	2.61
SK-MEL-28	−14.06	−3.55	−4.89	−4.92	−11.09	−4.63
SK-MEL-5	0.31	2.00	0.86	5.94	−3.74	−0.44
UACC-257	−8.15	−14.19	−18.68	17.45	−7.62	−1.41
UACC-62	N.T.	N.T.	N.T.	12.63	N.T.	N.T.
*Ovarian Cancer*						
IGROV1	5.29	11.14	6.71	24.43	1.54	27.56
OVCAR-3	−5.82	−2.81	−12.89	−8.39	−6.58	9.57
OVCAR-4	−8.19	−4.38	1.28	−0.80	−3.91	**53.12**
OVCAR-5	−10.42	−4.61	−1.61	−12.31	−0.50	4.92
OVCAR-8	−0.16	−4.64	−7.15	6.34	2.02	16.63
NCI/ADR-RES	−1.22	0.66	−3.45	−8.11	−6.46	5.13
SK-OV-3	−2.82	−5.01	−6.26	13.68	−16.74	13.87
*Renal Cancer*						
786-0	1.16	9.41	2.79	−0.09	0.56	21.33
A498	0.02	0.68	−3.11	4.71	5.97	−5.43
ACHN	−4.68	0.98	−3.79	4.11	−12.22	**60.41**
CAKI-1	N.T.	N.T.	N.T.	1.02	N.T.	N.T.
RXF 393	−11.41	−17.32	−13.02	−3.61	−13.69	−0.39
SN12C	−2.50	−1.40	−2.74	1.02	48.38	21.52
TK-10	−3.17	−4.14	−12.97	−23.0	9.04	−6.68
UO-31	11.67	11.34	14.20	11.83	8.66	25.50
*Prostate Cancer*						
PC-3	1.46	1.03	3.59	14.76	7.23	17.21
DU-145	−6.51	−7.26	−9.51	3.05	−8.97	−3.13
*Breast Cancer*						
MCF7	2.70	9.82	0.73	11.58	6.19	15.69
MDA-MB-231/**ATCC**	−9.78	−10.78	−9.11	10.81	−2.67	14.16
HS 578T	0.13	−4.94	−3.27	4.54	0.70	21.46
BT-549	−3.59	9.79	−17.43	−11.5	−8.83	−4.84
T-47D	0.23	4.18	−2.65	0.28	14.64	27.35
MDA-MB-468	−11.51	−13.19	−15.49	8.70	−14.97	−1.69

*NT: not tested.

Bold values signifies the growth inhibition parentage is higher than 50%.

**Table 2.** Growth inhibition percentages obtained from the single dose (10^−5^ M) test for compounds **8a, c, d, 9** and **10b, c**.

Panel/cell line	Compound					
	**8a**	**8c**	**8d**	**9**	**10b**	**10c**
*Leukemia*						
CCRF-CEM	2.31	−8.74	0.86	30.91	38.04	4.81
HL-60(TB)	22.48	25.51	11.84	11.79	1.69	−4.11
K-562	−3.68	11.36	−4.00	−3.06	24.18	−3.02
MOLT-4	5.79	7.19	3.49	22.66	38.29	7.03
RPMI-8226	2.00	−8.37	6.45	−6.21	7.52	1.29
SR	1.18	10.96	8.97	20.25	22.95	13.68
*Non-Small Cell Lung Cancer*						
A549/ATCC	−0.09	−3.31	−4.08	−5.98	2.36	−0.67
EKVX	19.50	20.55	7.21	17.03	12.38	10.04
HOP-62	−13.58	4.05	1.51	4.27	−6.39	0.38
HOP-92	9.27	15.93	4.73	13.39	39.54	40.41
NCI-H226	−8.48	7.59	5.22	15.47	−2.30	5.82
NCI-H23	0.28	20.68	9.95	19.05	9.39	14.25
NCI-H322M	5.51	3.90	1.50	23.88	14.39	13.49
NCI-H460	−8.23	−4.35	−4.87	−11.72	11.95	−3.23
NCI-H522	8.03	8.23	3.59	10.35	27.06	7.26
*Colon Cancer*						
COLO 205	−7.19	−1.97	−7.56	−11.74	−4.39	−6.78
HCC-2998	2.41	3.74	3.59	5.10	0.27	2.74
HCT-116	−7.69	1.69	7.42	3.99	−4.51	3.15
HCT-15	−2.04	−8.49	−5.55	−5.82	18.91	−5.30
HT29	7.56	5.97	−1.26	1.62	7.70	8.83
KM12	0.04	2.24	0.40	3.79	28.14	5.82
SW-620	−4.76	−3.16	−0.63	−8.79	−30.86	−11.68
*CNS Cancer*						
SF-268	−0.79	4.51	−2.90	25.55	29.01	6.35
SF-295	0.61	−0.93	−2.40	−3.92	−5.51	−2.66
SF-593	−8.46	3.89	8.43	−0.70	3.85	13.15
SNB-19	5.14	−4.77	2.02	11.34	6.67	−4.87
SNB-75	0.92	0.02	5.93	−0.56	17.50	19.53
U251	−1.70	−0.06	−2.50	9.85	5.64	0.92
*Melanoma*						
LOX IMVI	−2.33	4.93	0.28	13.28	18.99	9.33
MALME-3M	−10.46	4.22	5.14	19.00	−12.36	12.58
M14	−4.47	−0.94	3.95	−8.00	−2.10	2.73
MDA-MB-435	−2.24	−14.50	−6.10	−9.22	4.56	−2.02
SK-MEL-2	1.49	9.37	−1.69	18.31	10.15	−1.74
SK-MEL-28	−12.61	−3.74	−8.74	2.10	−7.04	1.30
SK-MEL-5	−0.67	5.22	−1.01	3.85	43.36	15.34
UACC-257	−5.90	−0.44	−6.35	−3.21	−14.11	0.35
UACC-62	N.T.	N.T.	N.T.	N.T.	N.T.	N.T.
*Ovarian Cancer*						
IGROV1	−3.29	25.56	9.45	23.49	20.55	26.65
OVCAR-3	−5.65	1.21	−8.47	32.23	24.19	−0.30
OVCAR-4	−10.71	−1.90	−1.77	17.82	9.65	−8.66
OVCAR-5	−3.95	−2.91	−7.96	−7.64	−30.85	−11.41
OVCAR-8	−5.87	3.66	1.33	1.29	3.64	−1.23
NCI/ADR-RES	−6.35	6.62	−3.70	3.79	4.30	−2.25
SK-OV-3	−7.07	12.56	3.69	7.98	0.81	7.06
*Renal Cancer*						
786-0	10.64	−2.04	−4.57	−3.72	11.35	1.85
A498	4.93	9.66	−0.85	1.48	−3.34	22.01
ACHN	−10.31	2.13	−0.34	2.07	7.34	4.01
CAKI-1	N.T.	N.T.	N.T.	N.T.	N.T.	N.T.
RXF 393	−15.68	−16.53	−9.45	5.47	4.90	3.39
SN12C	1.12	13.35	6.31	8.30	14.90	4.99
TK-10	9.75	−30.90	−47.95	−51.2	11.27	−36.27
UO-31	5.57	34.89	21.31	28.67	35.57	31.11
*Prostate Cancer*						
PC-3	5.06	11.35	12.47	13.76	27.02	16.52
DU-145	−7.70	−5.88	−11.21	−0.84	12.39	−4.94
*Breast Cancer*						
MCF7	9.07	3.10	1.60	11.52	14.72	1.96
MDA-MB-231/ATCC	−10.39	7.92	2.36	23.08	15.55	16.46
HS 578T	−4.78	3.74	5.99	−4.22	0.89	13.94
BT-549	3.96	−4.23	2.13	−10.8	9.63	−1.06
T-47D	−22.24	10.32	3.85	32.00	20.15	16.75
MDA-MB-468	−7.80	−3.83	0.37	18.48	8.05	−4.81

*NT: not tested.

### Detection of IC_50_ of compound 7d against lung cancer NCI-H460, NCI-H226, renal cancer ACHN and ovarian cancer OVCAR-4 cell lines

**Figure 3.**
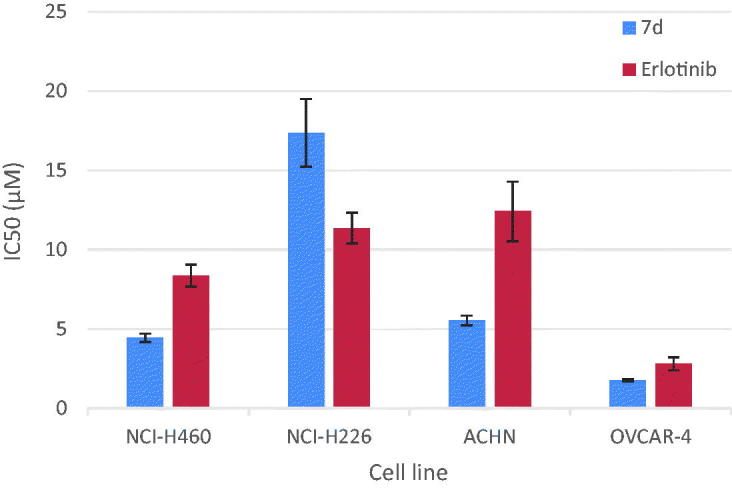
Graphical representation for concentrations required for 50% inhibition of cell viability (IC_50_) of compound **7d** compared to Erlotinib.

Compounds **7d** was selected to be further studied through determination of its half maximal inhibitory concentration (IC_50_) values against the most sensitive cancer cell lines compared to Erlotinib as a reference anticancer drug. The results of the mean values of experiments performed in triplicate were summarised in Table 3 and represented graphically in Figure 3. The *in vitro* results showed that the most sensitive cell line for compound **7d** was ovarian (OVCAR-4) with IC_50_ = 1.74 μM, which was 1.6 times more potent than Erlotinib. It also showed promising potent anticancer activity against renal (ACHN) cell line with IC_50_ value 5.53 μM representing 2.2 folds more potency than Erlotinib. Regarding lung cancer NCI-H460 cell line, compound **7d** (IC_50_ = 4.44 μM) was 1.9 folds more potent than Erlotinib. It is worthy mention that compound **7d** (IC_50_ = 18.73 μM) was almost 3.5 folds more potent than Elrlotinib against NCI-H460 EGFR-WT cell line. It possessed moderate anticancer activity against NCI-N226 cell line with IC_50_ = 17.36 μM. Structural activity relationship analysis revealed that anti-proliferative activity of the newly synthesised pyrazolo[3,4-*d*]pyrimidines correlates well with substitution pattern on position 4. It is worth noting that compound **7d**, a highly potent anticancer agent was among pyrazolopyrimidines **7a–d** having a phenyl amino group directly attached to the pyrimidine nucleus. Further analysis of these compounds clearly revealed the substitution pattern on this phenyl amino moiety had a notable effect on the anticancer activity. Grafting 3-Cl and 4-F substituents to the 4-anilino moiety enhanced the anti-proliferative activity against several tumor cell lines, otherwise 4-F and 4-CH_3_ substituents resulted in inactive members. Furthermore, the incorporation of the phenyl amino group through azomethine or piperazinyl linker regardless of the substitution pattern on the phenyl group abolished activity. Finally, replacement of the 4-anilino group with pharmacophoric moieties as carbonyl, amino, morpholine, 4-methylpiperazine or hydrazinyl groups or introducing additional amino group at C-6 position resulted in a dramatic loss in the anti-proliferative activity.

**Table 3.** Concentrations required for 50% inhibition of cell viability (IC_50_) of compound **7d** compared to Erlotinib.

Panel/ cell line		IC**_50_** (μM*±SD)	
		7d	Erlotinib
Lung cancer	NCI-H460	4.44 ± 0.26	8.36 ± 0.69
Lung cancer	NCI-H460 (EGFR-WT)	18.73 ± 0.71	64.54 ± 2.59
Lung cancer	NCI-H226	17.36 ± 2.13	11.36 ± 0.97
Renal cancer	ACHN	5.53 ± 0.31	12.41 ± 1.88
Ovarian cancer	OVCAR-4	1.74 ± 0.06	2.79 ± 0.41

*The results given are means of three experiments.

### Measurement of the effect of 7d on EGFR enzyme

**Figure 4.**
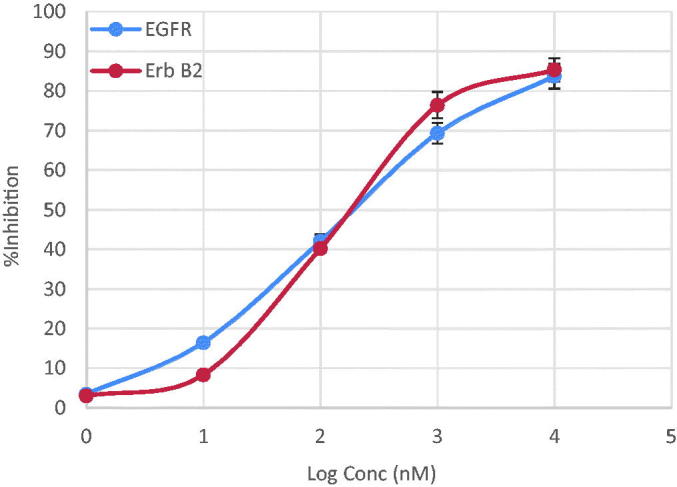
Line representation of the effect of compound **7d** on EGFR and ErbB2 tyrosine kinases.

We have investigated the inhibitory effect of compound **7d** on EGFR enzyme *in vitro*. The anti-proliferative activity of this compound appeared to correlate well with its ability to inhibit EGFR at sub-micromolar range with IC_50_ value 0.18 µM (Table 4 and Figure 4).

**Table 4.** IC_50_ values for the inhibitory activity of compound **7d** against EGFR and ErbB2 enzymes.

	IC_50_ (µM*±SD)	
Enzyme	**7d**	**Erlotinib**
EGFR	0.186 ± 0.730	0.030 ± 0.002
ErbB2	0.254 ± 0.640	0.115 ± 0.720

*The values given are means of three experiments.

### Measurement of the effect of 7d on ErbB2 enzyme

Since EGFR and ErbB2 receptors have the highest homology among the EGFR family members in their kinase catalytic domains and share many similar biochemical and kinetic properties. We have measured the inhibitory effect of compound **7d** on ErbB2 tyrosine kinase *in vitro*. Compound **7d** showed excellent inhibitory activity, at the sub-micromolar level with IC_50_ value 0.25 µM (Table 4 and Figure 4).

### Measurement of the effect of compound 7d on caspase-3 level

**Figure 5.**
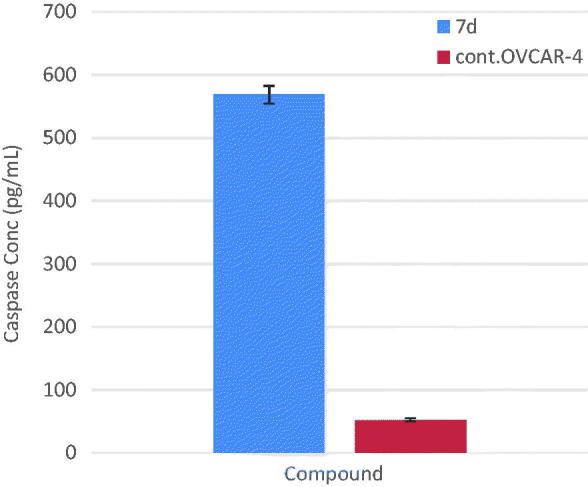
Graphical representation for active caspase-3 assay of compounds **7d**.

EGFR and ErbB2 tyrosine kinase inhibitors are well known to potentiate the intrinsic apoptotic pathway *via* increase in caspase-3 enzyme activity^34^^,^^35^. To evaluate the effect of compound **7d** on the level of active caspase-3, OVCAR-4 cells were treated with compound **7d** at its IC_50_ value for 24 h, before the enzyme assay. Compound **7d** resulted in a significant increase (almost 11 folds) in the level of active caspase-3 compared to control (Table 5 and Figure 5).

**Table 5.** Effect of compound 7d on active caspase-3.

Compound	Caspase3 conc. (Pg/mL*)
**7d**	568.50 ± 14.0
**Cont.OVCAR-4**	52.51 ± 2.46

*The values given are means ± SD of three experiments.

### Cell cycle analysis and detection of apoptosis

**Figure 6.**
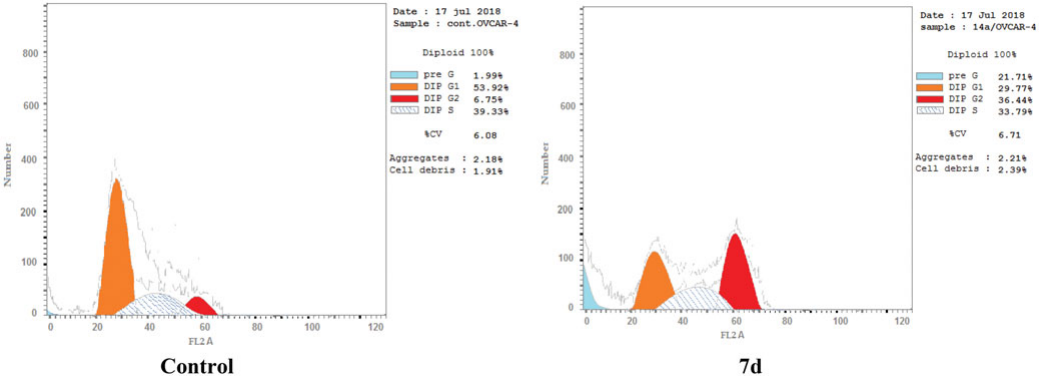
Effect of compound **7d** (1.74 μM) on DNA-ploidy flow cytometric analysis of OVCAR-4 cells after 24 h.

**Figure 7.**
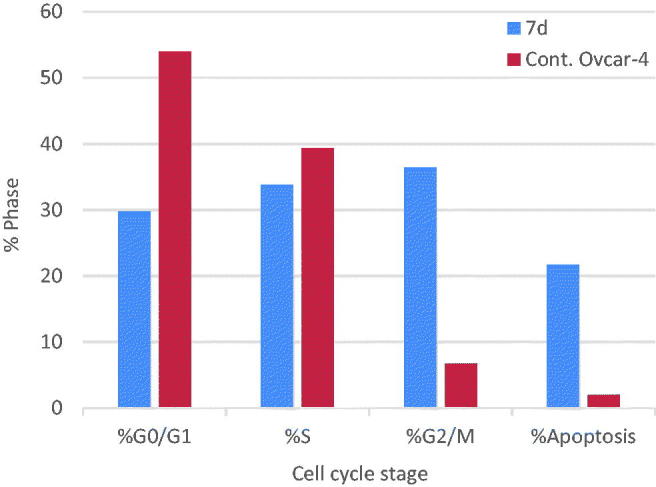
Graphical representation of the cell cycle analysis of compound **7d**.

The effect of the most active compound **7d** on the cell cycle progression and induction of apoptosis in OVCAR-4 cells was studied. OVCAR-4 cells were exposed to compound **7d** at its IC_50_ values for 24 h and its effect on the normal cell cycle profile and induction of apoptosis was analysed. Exposure of OVCAR-4 cells to compound **7d** resulted in an interference with the normal cell cycle distribution of this cell line. Interestingly, compound **7d** resulted in an increase in the cells accumulated in pre-G1 phase by almost 11-folds compared to control, the apoptotic activity was affirmed by the presence of a sub-G1 peak which may result from degradation or fragmentation of the genetic materials. Also, it showed significant increase in the percentage of cells at G2/M phases by 5 folds, compared to control (Table 6 and Figures 6, 7). These results suggest that compound **7d** exerted its cytotoxic activity by promoting cycle arrest at G2*/*M phase and apoptotic induction.

**Table 6.** Cell cycle distribution of compound 7d.

Compound	Cell cycle distribution			
	%G0/G1	%S	%G2/M	%Apoptosis
**7d**	29.77	33.79	36.44	21.71
**Cont.OVCAR-4**	53.92	39.33	6.75	1.99

### Apoptosis determination by Annexin-V assay

**Figure 8.**
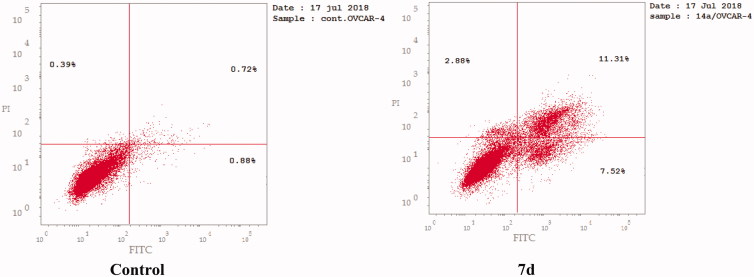
Representative dot plots of OVCAR-4 cells treated with **7d** (1.74 μM) for 24 h and analyzed by flow cytometry after double staining of the cells with annexin-V FITC and PI.

**Figure 9.**
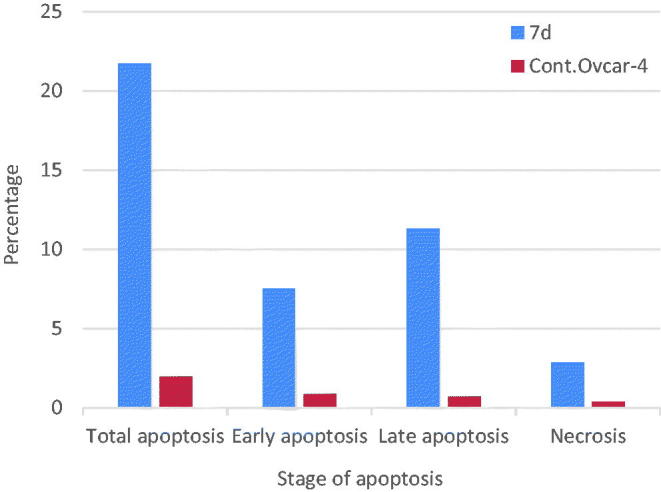
Graphical representation of effect of compound **7d** on apoptosis and necrosis.

The apoptotic activity of compound **7d** was further studied using Annexin V-FITC assay, which includes dual staining using Annexin V, a Ca^2+^-dependent protein, and Propidium iodide (PI). Annexin V binds to phosphatidylserine (PS) expressed only on the surface of the apoptotic cells and fluoresces green after interacting with the fluorochrome labelled annexin-V. On the other hand, PI stains DNA and enters only dead cells. This assay can give a differential analysis to the percentages of living, early apoptotic, late apoptotic and necrotic cells^36^. As shown in Figures 8, 9 and Table 7, after 24 h of treatment of OVCAR-4 cells with compound **7d** at its IC_50_ concentration a decrease in the percentage of the survived cells was observed. Moreover, a significant increase in the percentage of Annexin-V positive cells (almost 9 folds more than control) occurred indicating an early apoptosis (lower right quadrant). In addition, an increase by 7 folds more than control in the percentage of PI positive cells (upper left quadrant) indicating necrotic cells. Some treated cells were in a late apoptotic stage (upper right quadrant), this was indicated by the significant increase in the percentage of Annexin V positive, PI positive cells (16 folds more than control).

**Table 7.** Effect of compound **7d** on apoptosis and necrosis.

Compound	%Apoptosis			%Necrosis
	Total	Early	Late	
**7d**	21.71	7.52	11.31	2.88
**Cont.OVCAR-4**	1.99	0.88	0.72	0.39

## Conclusion

A series of novel pyrazolo[3,4-*d*]pyrimidines was synthesised. Twelve synthesised compounds were evaluated for their anticancer activity by NCI (USA). Compound **7d** exhibited potent anticancer activity at low concentrations. Pyrazolopyrimidine **7d** proved marked anticancer activity higher than Erlotinib. It showed 1.6-fold more potent anti-proliferative activity against ovarian (OVCAR-4) cell line with IC_50_ = 1.74 μM. It also exhibited promising potent anticancer activity against renal (ACHN) cell line with IC_50_ value 5.53 μM representing 2.2-fold more potency than Erlotinib. Regarding lung cancer NCI-H460 cell line, compound **7d** (IC_50_ = 4.44 μM) was 1.9 folds more potent than Erlotinib. It is worthy mention that compound **7d** was almost 3.5-fold more potent than Elrlotinib against NCI-H460 EGFR-WT cell line. It possessed moderate anticancer activity against NCI-N226 cell line with IC_50_ = 17.36 μM. The anti-proliferative activity of pyrazolopyrimidine **7d** appeared to correlate well with its ability to inhibit both EGFR and ErbB2 tyrosine kinases at sub-micromolar level with IC_50_ values 0.18 and 0.25 µM, sequentially. Dual inhibition of EGFR and ErbB2 enzymes leads to induction of the intrinsic pathway of apoptosis. This mechanistic pathway was confirmed by a significant increase in the level of active caspase-3, which is the key executer of apoptosis compared to the control (10.8 folds). Moreover, compound **7d** showed accumulation of cells in pre-G1 phase and annexin-V and propidium iodide staining in addition to cell cycle arrest at G2/M phase. Pyrazolo[3,4-*d*]pyrimidine **7d** is a privileged multi-targeted scaffold for the design and discovery of novel anticancer agents.
